# HELLP syndrome manifesting as abnormal fetal umbilical artery blood flow and rapidly worsening laboratory indexes: A case report

**DOI:** 10.1097/MD.0000000000031379

**Published:** 2022-11-04

**Authors:** Lin-bo Cheng, Qiang Wei, Li Zhang, Qin-yan Cao

**Affiliations:** a Department of Obstetrics and Gynecology, Chengdu Women’s and Children’s Central Hospital, School of Medicine, University of Electronic Science and Technology of China, Chengdu, China; b Department of Obstetrics and Gynecology, Key Laboratory of Birth Defects and Related Diseases of Women and Children of the Ministry of Education, West China Second Hospital, Sichuan University, Chengdu, China.

**Keywords:** diagnosis, HELLP syndrome, perinatal outcome, treatment

## Abstract

HELLP syndrome, a rare but serious obstetric complication, is often overdiagnosed due to its nonspecific symptoms and inappropriate clinical testing.

**Patient concerns:**

A 30-year-old nulliparous pregnant Chinese woman at gestational age of 28^+1^ weeks was admitted to our hospital because Doppler ultrasonography at a local hospital had detected loss of fetal umbilical artery end-diastolic blood flow lasting 12 hours. On admission to our hospital, the patient showed elevated blood pressure (148/84 mm Hg), but blood pressure and laboratory indicators after admission were normal. However, the patient developed abdominal pain during hospitalization.

**Interventions:**

Dexamethasone was given after admission to our hospital to promote fetal lung maturation, magnesium sulfate was given to protect fetal brain nerves, and maternal blood pressure was closely monitored. In addition, fetal umbilical artery blood flow was dynamically monitored. After three days in hospital with normal blood pressure, the patient developed abdominal pain accompanied by diarrhea. She was positive for Murphy’s sign and laboratory tests showed no obvious abnormalities. Acute cholecystitis was suspected, but symptomatic and supportive treatment did not relieve abdominal pain and her blood pressure increased progressively to 212/130 mm Hg. Magnesium sulfate was given immediately to prevent spasm, and nitroglycerin was administered intravenously against hypertension. Liver enzymes, blood coagulation, and routine urinalysis were abnormal. The patient was diagnosed with HELLP syndrome, and an emergency cesarean section was performed.

**Diagnosis:**

HELLP syndrome.

**Outcomes:**

After the cesarean section, platelet (PLT) count continuously decreased and transaminase and bilirubin levels continously increased. The newborn was transferred to the neonatal intensive care unit after birth and discharged at a corrected gestational age of 34 weeks. By postoperative day 6, laboratory indicators had returned to normal and the patient was discharged.

**Lessons subsections:**

Our case highlights that HELLP syndrome is a serious complication, and it should be diagnosed carefully and not arbitrarily on the basis of some abnormal indicators and stable clinical manifestations. Accurate early identification, active monitoring and management are essential for improving prognosis and avoiding maternal or infant mortality.

## 1. Introduction

HELLP syndrome, a rare but serious obstetric complication, is characterized by concurrent hemolysis, elevated liver enzymes and decreased platelet (PLT) count.^[1,2]^ The condition affects 0.2% to 1% of pregnant women.^[3–5]^ The syndrome is associated with poor prognosis, including higher risk of maternal morbidity and mortality.^[2]^ Early identification of HELLP syndrome, followed by active monitoring and management are key to improving prognosis for mother and infant.^[2,3]^

As a result of its nonspecific symptoms, the syndrome is overdiagnosed, leading to unnecessary tests and untimely termination of pregnancy. Clinicians may fail to perform the appropriate laboratory tests in a timely manner, delaying detection of the syndrome.

Here we describe a case of HELLP syndrome that highlights the importance of early diagnosis and active management in order to achieve good outcomes for mother and fetus.

## 2. Case report

A 30-year-old nulliparous pregnant Chinese woman (G2P0 + 1) at gestational age of 28^+1^ weeks was admitted to our hospital because Doppler ultrasonography at a local hospital had detected loss of fetal umbilical artery end-diastolic blood flow lasting 12 hours. Before admission to our hospital, she showed a normal result on the fetal nuchal translucency test, and a second-trimester screening test for Down syndrome suggested low risk. Ultrasonography suggested no obvious abnormalities in the fetus. The patient was diagnosed with gestational diabetes mellitus in the second trimester based on the 75-g oral glucose tolerance test, at which time she received nutritional guidance. The patient indicated that since then, she did not regularly monitor blood glucose.

On admission to our hospital, the patient had a body temperature of 36.5 ℃, heart rate of 55 beats/min, respiration rate of 20 breaths/min, and blood pressure of 148/84 mm Hg. Cardiopulmonary auscultation was normal. Obstetric examination revealed an abdominal circumference of 96 cm, uterine height of 24 cm, fetal heart rate of 140 beats/min, and no palpable contractions. Ultrasonography showed the fetus to be in the breech position; double parietal diameter was 6.6 cm, head circumference was 24.8 cm, femur length was 4.6 cm, and fetal abdominal circumference was 22.4 cm. These diameter measurements were less than the 10th percentile for fetuses of the same gestational age, indicating fetal growth restriction. The amniotic fluid index was 13.5 cm, no end-diastolic blood flow signal was detected in the umbilical artery, and the A-wave of the venous catheter was not missing or reversed.

The patient’s urine on admission scored initially 2+ for protein but was subsequently negative on retesting (Table 1). Routine analysis of blood, coagulation function as well as liver and kidney function were normal. On the basis of these findings and the patient’s medical history, she was initially diagnosed with gestational diabetes mellitus, potential gestational hypertension, fetal growth restriction, and abnormal umbilical arterial flow. In light of the fetal growth restriction, the patient was offered amniocentesis to test for genetic abnormalities, but she refused.

**Table 1:** Perioperative laboratory indexes of the patient.

	WBC(×10^9^/L)	HGB(g/L)	PLT(×10^9^/L)	FIB(g/L)	ALT(U/L)	AST (U/L)	TB(μmol/L)	DBIL(μmol/L)	IBIL (μmol/L)	LDH(U/L)	Urea mmol/L	Cr mmol/L	Urinary protein	Occult blood	24h urinary protein(g)
April 6th 22:00 (day of admission)	8.89	111	278	3.51	12.2	16	5.2	1.2	4	199	3.55	33.9	2+	-	
April 7th 09:00 (day 1 after admission)													(-)	(-)	
April 9th 06:00 (day 3 after admission)	12	114	270		35.3	44.9	4.3	1.2	3.1						
April 9th 11:00	16.5	107	153	1.87	297	396	19.7	4.1	15.6	1140	5.41	34.6	4+	3+	
April 9th 16:00 (operative day)	Tip blood coagulation			1.16	287	629	34.7	9.5	25.2	1150	4.40	29.6	2+	3+	
April 9th 19:00	12	94	57	1.88	257	616	42.8	15.9	26.9	1155	5.18	41.0			1.89
April 10th 07:00 (Postoperative day 1)	8.8	84	35	2.70	178	185	35.4	15	20.4	1099	4.68	43.0			
April 11th 07:00 (Postoperative day 2)	13.7	87	52	4.44	131	63	13.4	3.6	9.8		2.44	37.2			
April 12th 07:00 (Postoperative day 3)	12.9	88	85		89	24	10.3	2.0	8.3	373	4.22	39.0			0.12
April 15th 07:00 (Postoperative day 6)	8.12	98	231		40	16.5	7.1	2.0	5.1		4.56	36.5			

ALT = alanine aminotransferase, AST = aspartate aminotransferase, Cr = creatinine, DBIL = direct bilirubin, FIB = Fibrinogen, HB = hemoglobin, IBIL = indirect bilirubin, LDH = lactate dehydrogenase, PLT = platelet, TB = total bilirubin, WBC = white blood cell.

The patient was administered dexamethasone (6 mg Q 12 h) via intramuscular injection to promote fetal lung maturation, and she was given magnesium sulfate drops intravenously to protect the fetal brain. The patient’s blood pressure was closely monitored, and fetal umbilical artery blood flow was dynamically monitored. On day 2 after admission, the patient and family members requested that the pregnancy be terminated, so the patient was prepared for induction of labor. Magnesium sulfate was discontinued. During the two days after admission, the patient showed normal blood pressure (102‐133/62‐85 mm Hg).

Early in the morning on day 3, the patient reported abdominal pain and one episode of diarrhea. Blood pressure was normal. Physical examination revealed painful face, passive position, tenderness in the right upper quadrant of the ribs, and the presence of Murphy’s sign, but no tenderness around the umbilical cord, no obvious tension in abdominal muscles, and no tenderness at McIntosh’s point. Routine blood analysis and tests of liver function and blood amylase were normal (Table 1). Color Doppler ultrasonography of the liver, bile, pancreas and spleen revealed no obvious abnormalities.

Acute cholecystitis was suspected, and the patient was given symptomatic and supportive treatment: fasting, atropine (0.5 mg) against intramuscular spasm, intravenous sodium cefoperazone sulbactam (2 g Q 8 h) against infection, as well as supplementation with fluid and potassium. During these treatments, the abdominal pain persisted but moved gradually to the subxiphoid process. Within 2.5 h, blood pressure increased from 148/98 mm Hg to 212/130 mm Hg. Magnesium sulfate was immediately given to prevent spasm, pethidine hydrochloride for sedation and analgesia, and intravenous nitroglycerin against hypertension. After placement of the retention catheter, the urine was found to be dark brown. Analyses of blood, urine, urinary amylase, coagulation, D-dimer, liver and kidney function, electrolytes, and myocardial damage were urgently performed. These tests showed that alanine aminotransferase (ALT) had increased to 297 U/L, aspartate aminotransferase (AST) to 396 U/L, and lactate dehydrogenase (LDH) to 1140 U/L; conversely, fibrinogen had decreased to 1.87 g/L (Table 1). PLT count as well as total, direct and indirect levels of bilirubin had changed substantially from their values 5 hours previously, though they remained within the normal range. Urinalysis indicated a score of 4+ for urinary protein and 3+ for occult blood.

HELLP syndrome was suspected. We advised the patient against vaginal delivery in light of her rapidly deteriorating condition and her cervical immaturity based on BISHOP scoring. The patient consented to an emergency cesarean section on day 3 after admission. After an uneventful procedure, a live male baby was born with a weight of 765 g, length of 30 cm, and Apgar scores of 5 at 1 minute, 8 at 5 minutes, and 8 at 10 minutes. Amniotic fluid had a volume of 400 mL and tawny color. The patient lost about 500 mL of blood during the procedure.

After surgery, she was transferred to the intensive care unit, where treatment with magnesium sulfate and cefoperazone sulbactam were continued. PLT count decreased continuously to 35 × 10^9^/L, while transaminase and bilirubin levels increased continuously. By day 6 after surgery (day 9 after admission), laboratory indicators had returned to normal and the patient was discharged.

## 3. Ethic statement

The requirement for institutional review board approval was waived owing to the retrospective nature of the study. Written informed consent was obtained from the patient for the publication of this case report.

## 4. Discussion

HELLP syndrome can be a serious complication of hypertensive disorders during pregnancy. It can occur before the onset of clinical symptoms of preeclampsia, and it can occur in women suffering antiphospholipid syndrome.^[2]^ Up to 70% of cases of HELLP syndrome occur at gestational ages between 28 and 36 weeks, and 30% of cases occur during the postpartum period.^[6]^ The syndrome in our patient occurred at 28+ weeks of gestation.

HELLP is typically diagnosed based on laboratory tests, but consensus on diagnostic criteria is lacking. Guidelines from the American College of Obstetricians and Gynecologists^[7,8]^ suggest that all three of the following should be present: LDH ≥ 600 U/L, AST or ALT levels that exceed 2 times the upper limit of the normal reference range, and PLT count < 100 × 10^9^/L. In addition, the guidelines emphasize that maternal and fetal condition can abruptly and progressively worse. Our patient satisfied the three criteria and maternal condition indeed worsened abruptly.

The most frequent symptoms of HELLP syndrome include proteinuria, hypertension, right upper abdominal pain, nausea/vomiting, headache, altered vision, and jaundice.^[9]^ Hypertension and proteinuria occur in many, but not all, cases of HELLP syndrome.^[2]^ Our patient had symptoms of hypertension, proteinuria and right upper abdominal pain. Our case highlights that HELLP syndrome is a serious complication, and it should be diagnosed carefully and not arbitrarily on the basis of some abnormal indicators and stable clinical manifestations.

In fact, the guidelines from the American College of Obstetricians and Gynecologists recommend laboratory tests at least every 12 hours for HELLP patients.^[7]^ We performed postoperative tests only about every 24 hours, which was inadequate, especially in light of the fact that the patient’s PLT count dropped as low as 35 × 10^9^/L. Close monitoring is important for avoiding serious complications. In addition, we did not monitor the patient’s kidney function after her cesarean section, even though HELLP syndrome can involve microvascular dysfunction in the kidney.^[10]^

Our case highlights the challenge in detecting HELLP syndrome. Blood pressure was normal after admission, and urinalysis was negative for protein (upon retest). After the onset of abdominal pain, urinalysis showed hemolysis, with the dark tawny color of the urine clearly indicating hemoglobinuria. This clinical presentation is consistent with a report that in HELLP syndrome, pain may appear several hours before abnormal laboratory test results.^[11]^ In fact, laboratory tests only 2 hours after onset of abdominal pain in our patient were not abnormal for hemoglobin (HB) or PLT count, and AST was only slightly elevated. At 7 hours after the onset of abdominal pain, her laboratory indicators showed significantly elevated ALT and AST and slightly reduced levels of HB and PLTs, consistent with the suggestion that hematological tests at 4 to 6 hours after onset of symptoms can help diagnose HELLP^.[11]^

The only treatment completely effective for HELLP syndrome is pregnancy termination.^[7]^ According to the patient’s condition, PLT transfusion, adrenal corticosteroids, plasma exchange or hemodialysis may be required. HELLP syndrome is not normally an indication for cesarean section, but the immature cervical condition in our patient as well as her unstable vital signs led us to recommend this intervention.

We gave our patient dexamethasone to protect PLTs. A Cochrane systematic review concluded that glucocorticoids can improve PLT count without increasing risk of maternal or perinatal death.^[12]^ Whether corticosteroids can mitigate HELLP syndrome itself remains unclear.

Our patient showed severe clinical manifestations consistent with HELLP syndrome, and all perioperative laboratory indexes sharply deteriorated initially but then recovered to normal levels. If, however, PLT count continues to decrease while liver enzymes continue to increase beyond postpartum day 4, diagnoses other than HELLP syndrome should be considered, including thrombocytopenic purpura, hemolytic uremic syndrome, acute fatty liver in pregnancy, antiphospholipid syndrome, and systemic lupus erythematosus.^[2,9]^

Our case highlights the need for considering HELLP syndrome in a timely fashion, in particular through careful monitoring of laboratory results in order to detect abrupt deterioration. Fetal lung maturity should be promoted, pregnancy should be actively terminated and laboratory indicators, including renal function, should be monitored every 12 hours after diagnosis until they return to normal. Symptomatic treatment should be given to reduce the risk of serious complications in mother or child.

## Author contributions

Lin-bo Cheng drafted the manuscript, collected and analyzed the data. Qiang Wei conceived and designed the study, helped draft the manuscript, revised it critically for important intellectual content, and coordinated data collection. All authors read and approved the final manuscript.

**Conceptualization:** Lin-bo Cheng, Li Zhang, Qiang Wei.

**Data curation:** Lin-bo Cheng, Qin-yan Cao.

**Writing—original draft:** Lin-bo Cheng, Qiang Wei.

**Writing—review & editing:** Qiang Wei, Li Zhang.

**Writing—review and language polish:** Qiang Wei, Li Zhang.
